# Molecular Mechanism of YuPingFeng in the Treatment of Asthma Based on Network Pharmacology and Molecular Docking Technology

**DOI:** 10.1155/2022/7364126

**Published:** 2022-09-05

**Authors:** Li Shen, Jinmiao Lu, Guangfei Wang, Cheng Wang, Zhiping Li

**Affiliations:** ^1^Department of Pharmacy, The Affiliated Suzhou Science & Technology Town Hospital of Nanjing Medical University, Suzhou, Jiangsu 215153, China; ^2^Department of Clinical Pharmacy, Children's Hospital of Fudan University, National Children's Medical Center, Shanghai 201102, China; ^3^The Health Supervision Institute of Suzhou High-Tech Zone, Suzhou, Jiangsu 215007, China

## Abstract

**Objective:**

To explore the molecular targets and mechanism of YuPingFeng (YPF) for the treatment of asthma by using network pharmacology and molecular docking.

**Methods:**

The potential active ingredients and relevant targets of YPF were obtained from the Traditional Chinese Medicine Systems Pharmacology Database and Analysis Platform (TCMSP). Asthma-related gene targets were retrieved from GeneCards, OMIM, DrugBank, PharmGKB, and TTD databases. The protein-protein (PPI) network between YPF and asthma common targets was constructed by SRING online database and Cytoscape software. GO and KEGG analyses were performed to explore the complicated molecular biological processes and potential pathways. Finally, a molecular docking approach was carried out to verify the results.

**Results:**

We obtained 100 potential targets of the 35 active ingredients in YPF and 1610 asthma-related targets. 60 YPF-asthma common targets were selected to perform PPI analysis. Seven core genes were screened based on two topological calculation methods. GO and KEGG results showed that the main pathways of YPF in treating asthma include TNF signaling pathway and PI3K-Akt signaling pathway. Finally, the molecular docking results indicated that the key ingredients of YPF had a good affinity with the relevant core genes.

**Conclusion:**

This study reflects the multicomponent, multitarget, and multipathway characteristics of YPF in treating asthma, providing a theoretical and scientific basis for the intervention of asthma by traditional Chinese medicine YPF.

## 1. Introduction

Asthma is a complex chronic airway inflammatory disorder characterized by recurrent wheezing, shortness of breath, and chest tightness, which usually starts in childhood [1, 2]. The disease presents a global healthcare burden, with more than 300 million people suffering from asthma globally [3]. Standardized asthma treatments are vital for improving quality of life. Currently, inhalant therapy is performed as the main treatment for asthma control, including inhaled glucocorticoids (ICS), *β*2-receptor agonist, and M-receptor blockers [4, 5]. However, fear of suspicious side effects from some inhaled drugs, especially ICS, leads to poor adherence and is associated with poor asthma control [6, 7]. Biological preparations such as omalizumab were introduced as severe asthma treatments, but the strict clinical indications and the relatively high price limit the application [8–10]. Therefore, a safer and more effective therapeutic regimen or alternative therapy for asthma is still needed.

Traditional Chinese medicine (TCM) has a long history. In recent years, great attention has been focused on TCM because of its relative safety and unique superiority. TCM such as Xin Guan-1 Formula and LianHuaQingWen Capsule had significant beneficial effects in treating patients infected with COVID-19 [11, 12]. YuPingFeng (YPF) is a classical TCM that comes from famous doctors of the Yuan Dynasty in China. The YuPingFeng formula consists of three herbs: Radix Astragali (Huang Qi (HQ)), Rhizoma Atractylodis Macrocephalae (Bai Zhu (BZ)), and Radix Saposhnikoviae (Fang Feng (FF)). A previous study [13] found that YPF is effective for treating chronic obstructive pulmonary disease (COPD), and the researchers explored the potential mechanisms behind the curative effects of the drug based on network pharmacology technology. A recent study has indicated that YPF is beneficial for relieving the relapse of asthma induced by house dust mites [14]. COPD and asthma are linked diseases; therefore, understanding the relationship between targets and the mechanism of YPF against asthma would be worthy of research.

**Figure 1:**
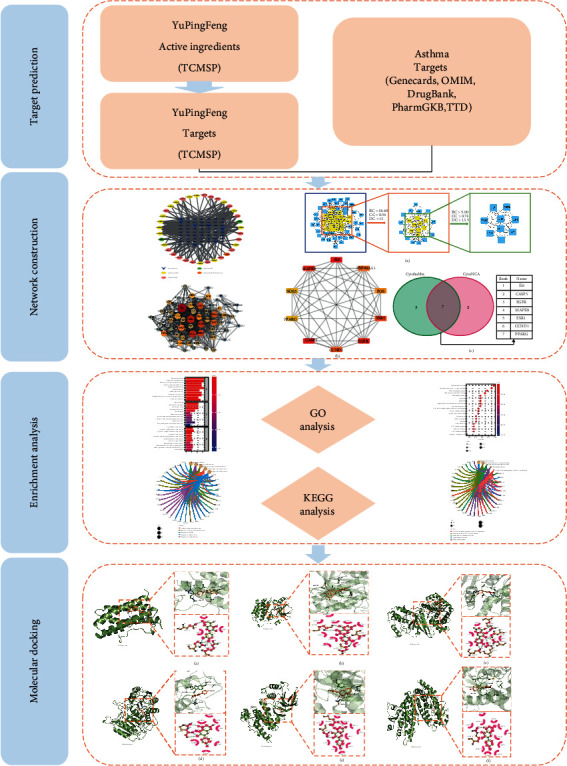
Flowchart of network pharmacology and molecular docking.

Network pharmacology is an emerging discipline that combines bioinformatics, molecular biology, and traditional pharmacology [15]. It conducts systematic analysis by constructing a “drug-gene-target-disease” interaction network and reveals the synergistic interaction mechanism of the drug against disease. Based on the scientific strategy of network pharmacology, the current research is aimed at systematically exploring the relevant targets and potential signaling pathways of YPF against asthma. The entire workflow of this study is shown in Figure 1.

## 2. Materials and Methods

### 2.1. YPF Active Ingredients and Targets

We obtained the YPF active ingredients and the related targets from the Traditional Chinese Medicine Systems Pharmacology Database and Analysis Platform (TCMSP) (https://tcmsp-e.com/) [16], which is an open database of Chinese herbal medicines and shows the relationships between drugs, targets, and diseases. The active ingredients of the YPF herbs, including “Huang Qi,” “Bai Zhu,” and “Fang Feng,” were screened with the conditions oral bioavailability (OB)≧30% and drug-likeness (DL)≧0.18 [17]. The protein targets related to active ingredients were also retrieved from TCMSP. Afterward, all the obtained targets were standardized with the UniProt database (https://www.uniprot.org/). Finally, valid gene symbols were obtained after removing mismatches and redundant duplicates.

### 2.2. Prediction of Asthma-Related Target Genes

Targets relevant to asthma were collected from five databases with the keyword “asthma”: the Human Gene Database (https://www.genecards.org/, GeneCards), Online Mendelian Inheritance in Man (https://omim.org/, OMIM), DrugBank Online (https://go.drugbank.com/, DrugBank), Pharmacogenomics Knowledgebase (https://www.pharmgkb.org/, PharmGKB), and Therapeutic Target Database (http://db.idrblab.net/ttd/, TTD). The union of all the search results was used to establish an asthma-related gene set, and the set was visualized by R 3.6.3 software.

### 2.3. Network Construction

#### 2.3.1. Construction of an Ingredient-Target Network

The active ingredients of YPF and the asthma-related genes were taken as an intersection. Then, the ingredient-target network was established by Cytoscape v3.8.0 software.

#### 2.3.2. Construction of Protein-Protein Interaction (PPI) Network

Overlaps between YPF-related targets and asthma-related targets were obtained to clarify the interaction between drugs and disease. These overlaps were put into the STRING database (https://string-db.org/) to construct the PPI network. Parameters were set to “Homo sapiens” in the organism and the minimum required interaction score cutoff set at 0.400. Disconnected nodes were hidden in the network. Then, the PPI network was constructed, and the result was visualized by Cytoscape v3.8.0 software.

#### 2.3.3. Identification of Core Genes

To analyze further into the PPI network, two approaches were followed to screen core genes. In the first approach, core genes were screened with topological properties by CytoNCA plugin in Cytoscape. Three parameters, “Betweenness Centrality (BC),” “Closeness Centrality (CC),” and “Degree Centralities (DC),” were selected to calculate the gene scores. Genes with score values higher than the median value were obtained. To identify crucial core genes, the filter process was performed twice. A second approach involves using the Cytohubba plugin of the Cytoscape software. We used the plugin to select the top 10 genes based on a Maximum Neighborhood Component (MNC) calculation method. The common targets set from both ways were regarded as the core genes.

### 2.4. GO and KEGG Enrichment Analysis

To analyze the molecular biological functions of YPF-asthma common targets, Gene Ontology (GO) and Kyoto Encyclopedia of Genes and Genomes (KEGG) analyses were performed. The GO enrichment analysis involved three main categories: biological process (BP), cellular component (CC), and molecular function (MF). KEGG enrichment analysis was utilized for revealing complicated biological pathways. R 3.6.3 software was used to perform both GO and KEGG analysis, with the screening criterion as the following filters: *q* value < 0.05.

### 2.5. Molecular Docking

Molecular docking simulation technology was used to predict molecular targets of YPF for treating asthma. The 2D structures of small molecule ligands were downloaded from the PubChem database (https://pubchem.ncbi.nlm.nih.gov/). Conversion of 2D structures into 3D formats with minimum energy was performed by ChemBio 3D software. The forms of protein receptors with the screen criteria of “Homo sapiens,” “X-ray Diffraction,” and “Protein” were downloaded from the RCSB Protein Data Bank database (PDB, https://www1.rcsb.org/). The ligands and the receptors were prepared in PyMOL v2.4.0 and AutoDock v1.5.6 by removing water molecules, adding hydrogen atoms and charges. Afterward, AutoDock Vina was used to performing molecular docking. The results were analyzed by the Protein-Ligand Interaction Profiler (PLIP) web tool, and finally, the output results were visualized using LigPlot v2.2.4 and PyMOL software.

## 3. Results

### 3.1. Screening of Active Ingredients and Targets

**Figure 2:**
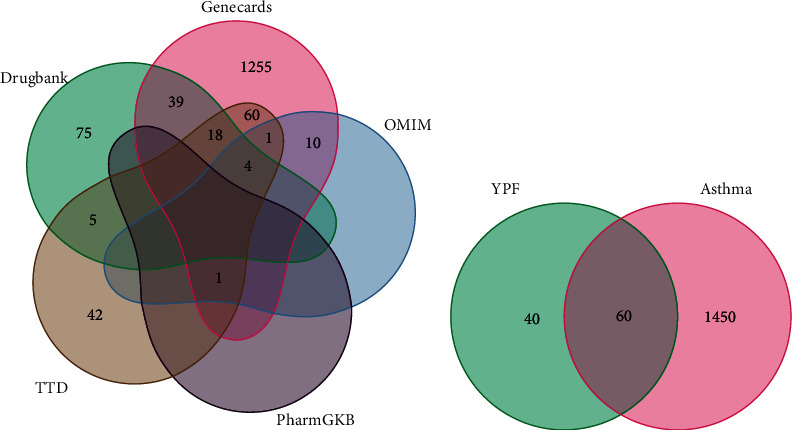
Identification of active ingredients and targets. (a) Identification of the asthma-related genes from five online databases. (b) Identification of YPF-asthma common targets.

With the screen criteria of OB≧30% and DL≧0.18, a total of 45 active ingredients were obtained, of which 20 were from HQ, 7 from BZ, and 18 were from FF. At the same time, 716 YPF-related targets were identified based on the TCMSP database. After removing redundant ones and standardized by UniProt, we finally recognized 100 potential targets of the 35 active ingredients for further analysis. Detailed information on these active compounds is listed in Supplementary Table S1. These ingredients were essential to the treatment of related disorders through a synergistic action. Besides, we obtained 141, 1388, 16, 1, and 131 asthma-related genes from DrugBank, GeneCards, OMIM, PharmGKB, and TTD databases, respectively (Figure 2(a)). By comparing the YPF-related genes and asthma-related genes, we found that asthma shares 60 targets with YPF (Figure 2(b)). These 60 common targets were obtained for analysis in the next step.

### 3.2. Common Target-Active Ingredient Network

**Figure 3:**
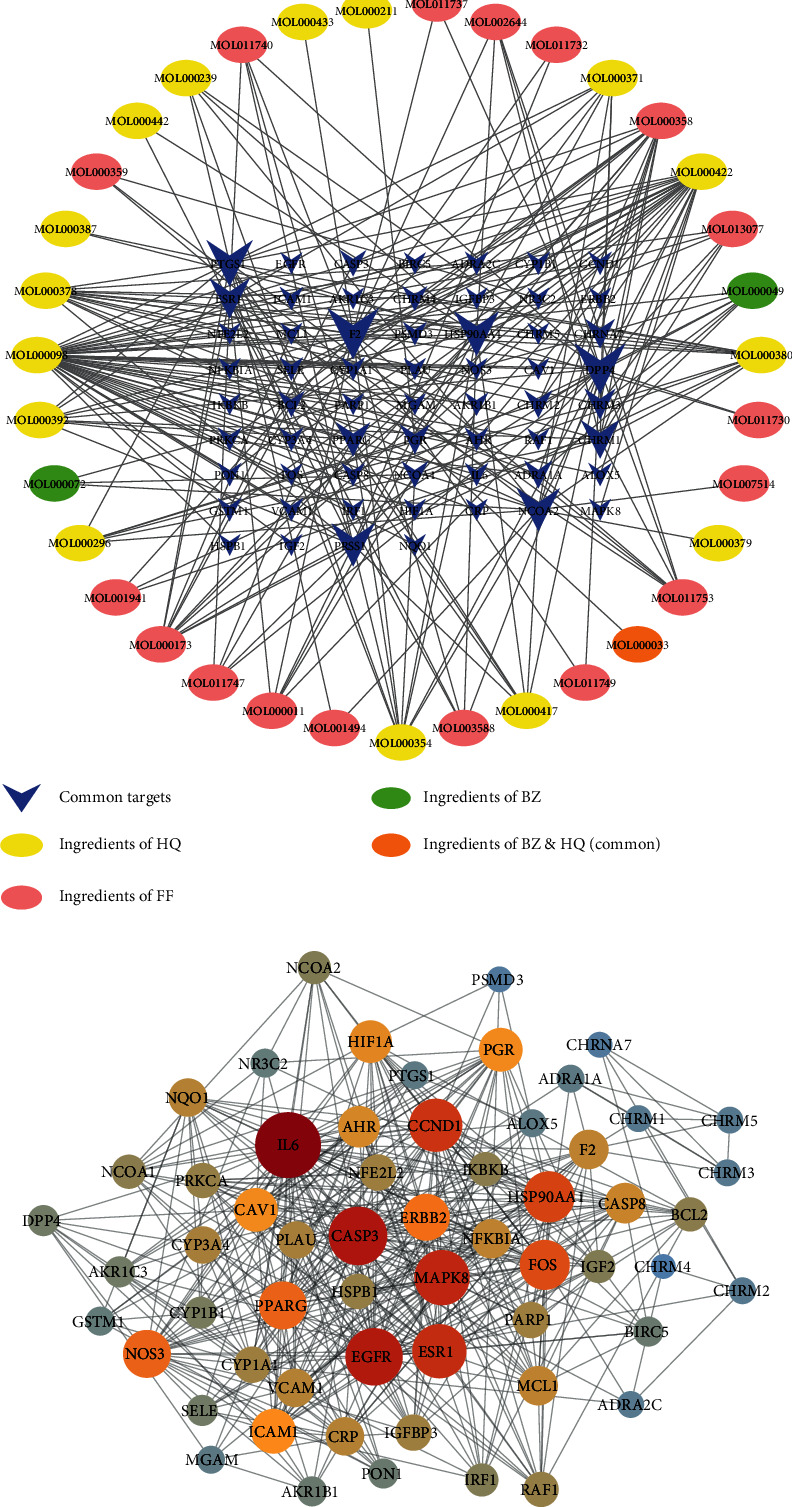
Network construction. (a) Compound-target network. The blue nodes represent drug-disease common targets. The larger the node, the more crucial it is. The yellow nodes represent active ingredients of Huang Qi (HQ). The pink nodes represent active ingredients of Fang Feng (FF). The green nodes represent active ingredients of Bai Zhu (BZ). The orange nodes represent the common active ingredients of the BZ and HQ. (b) PPI network. The darker the color, as well as the larger the node, the more significant it is.

A compound-target network was constructed by linking the active ingredients of YPF with their targets. We visualized the network with 95 nodes and 240 edges by Cytoscape 3.8.0. (Figure 3(a)). Calculated with Analyze Network plugin and based on degree, the top five significant ingredients of YPF were quercetin, kaempferol, beta-sitosterol, 7-O-methylisomucronulatol, wogonin, and (6aR,11aR)-9,10-dimethoxy-6a,11a-dihydro-6H-benzofurano[3,2-c]chromen-3-ol.

### 3.3. PPI Network

PPI network analysis on the asthma targets was performed by STRING online database, and the results were visualized by Cytoscape software. The PPI network of these related targets was built with 59 nodes and 441 edges, and the average node degree was 15 (Figure 3(b)). In this PPI network, nodes represent proteins, and edges represent protein-protein associations.

### 3.4. Identification of Core Genes

**Figure 4:**
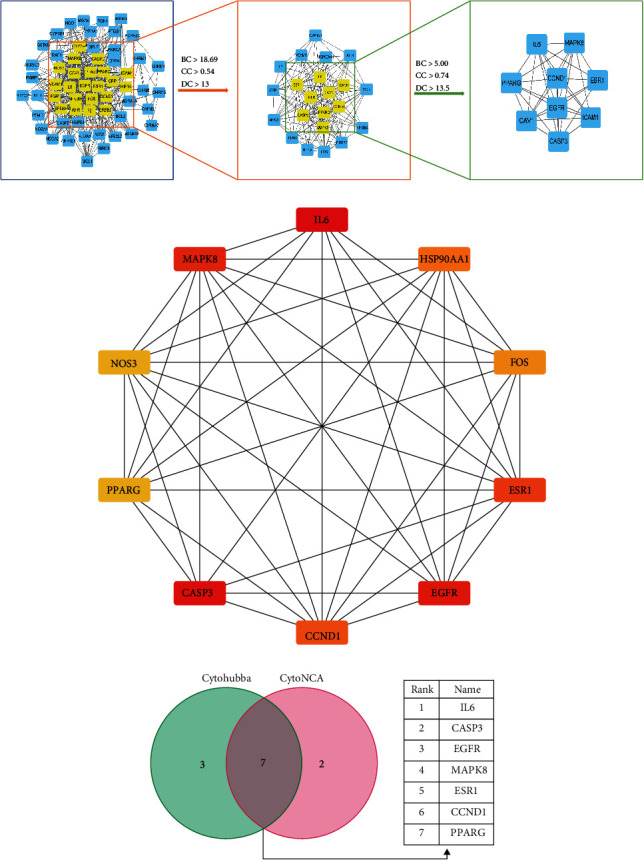
Identification of core genes. (a) Identification by CytoNCA. (b) Identification by Cytohubba. (c) Screening of core genes by taking an intersection.

The CytoNCA plugin in Cytoscape was used for topological analysis of the targets in the PPI network. In this study, the topological analysis was based on three topological parameters: Betweenness, Closeness, and Degree. After the first filter process, screening with BC > 18.69, CC > 0.52, and DC > 13, 22 nodes with 151 edges were obtained. In the second filter process screening with BC > 5.00, CC > 0.74, and DC > 13.5, finally, we identified 9 highly connected nodes (Figure 4(a)). Besides, the Cytohubba plugin of the Cytoscape software was used to analyze the top 10 genes based on the MNC method (Figure 4(b)). The Venn diagram (Figure 4(c)) showed the intersection of two gene sets, and seven core genes were obtained: IL6, CASP3, EGFR, MAPK8, ESR1, CCND1, and PPARG.

### 3.5. GO and KEGG Enrichment Analysis

**Figure 5:**
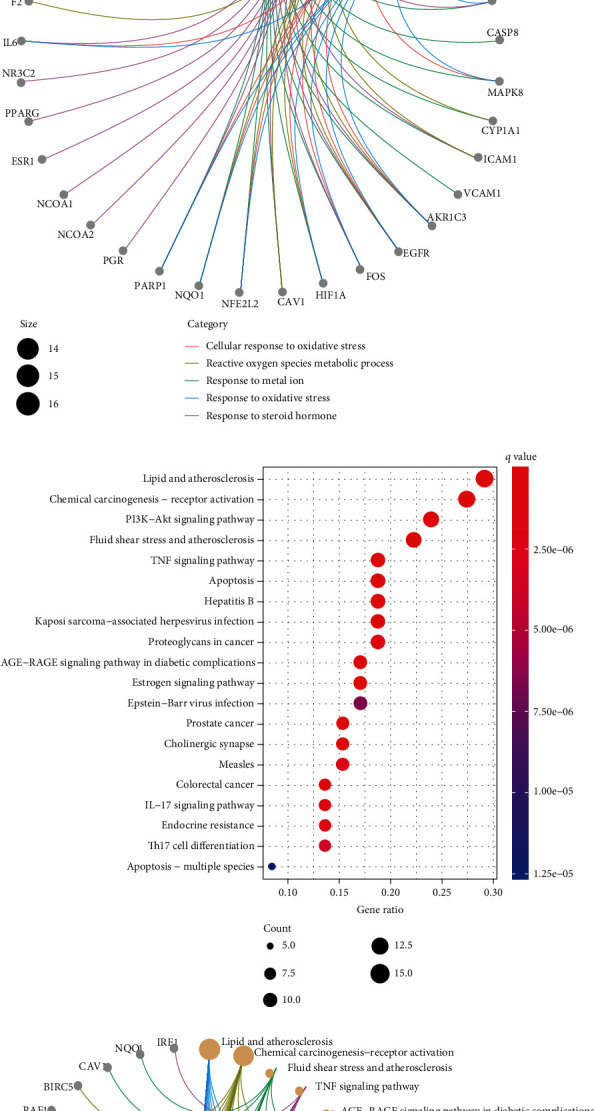
GO and KEGG analysis of asthma-related genes. (a) The bar diagram showed the top ten significantly enriched terms in BP, CC, and MF, respectively. (b) The top five BP terms and the related genes. (c) The bubble diagram of the top 20 KEGG pathways. Gene ratio represents the enriched genes to all genes. The count represents the number of enriched genes. (d) The top five KEGG pathways and related genes.

To further explore the effector mechanism of YPF in treating asthma, the drug-disease common targets were analyzed by the R package “cluster profiler” to perform GO and KEGG analysis. In the GO functional enrichment, common targets were annotated in three parts: BP, CC, and MF. We obtained 1312 GO enrich results with confidence levels of *p* value < 0.05 and *q* value < 0.05. A total of 1158 terms about biological processes were selected, mainly including response to metal ion (GO:0010038), response to steroid hormone (GO:0048545), reactive oxygen species metabolic process (GO:0072593), cellular response to oxidative stress (GO:0034599), and response to oxidative stress (GO:0006979) (Figures 5(a) and 5(b)). A total of 60 terms about cellular components were obtained, including membrane raft, membrane microdomain, and membrane region. Meanwhile, 94 molecular function terms would be related to acetylcholine receptor activity, G protein-coupled amine receptor activity, and G protein-coupled serotonin receptor activity. KEGG pathway enrichment analysis was carried out to indicate signaling pathways in connection with the antiasthma effect of YPF. A total of 125 pathways were meaningfully enriched, and the 20 top-rank pathways were screened out (Figures 5(c) and 5(d)). Particularly, based on literature research reports [18–20] and the KEGG results, the potential mechanism of YPF in treating asthma was mainly focused on the TNF signaling pathway, PI3K-Akt signaling pathway, IL-17 signaling pathway, and Th17 cell differentiation (Table 1). The targets of YPF active ingredients were enriched in different pathways, interacting and coordinating with each other.

### 3.6. Validation by Molecular Docking

**Figure 6:**
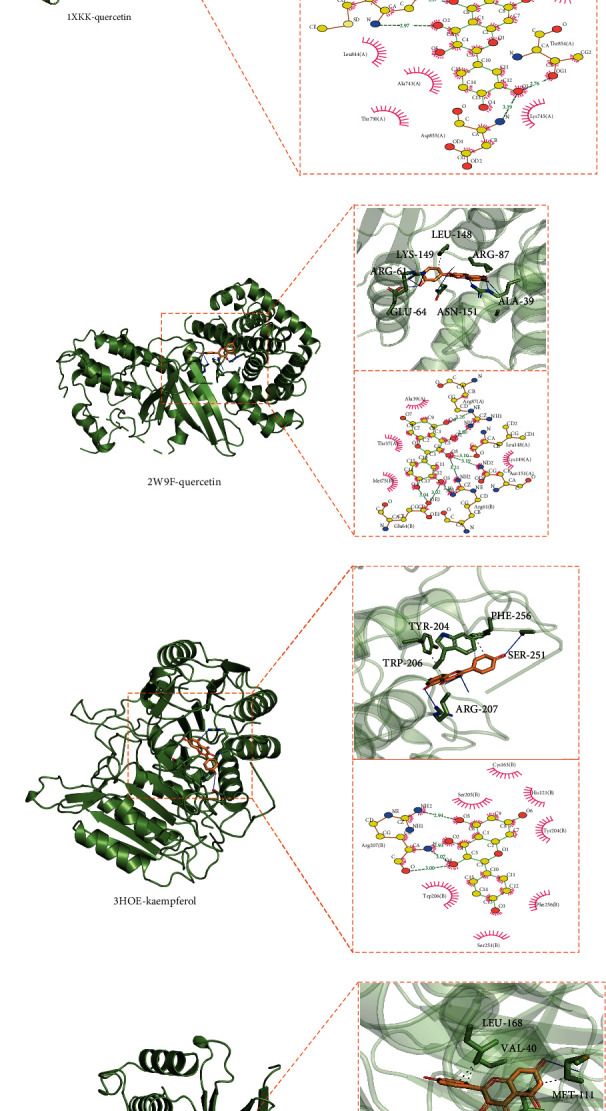
Molecular docking of active ingredients and core genes: (a) IL6-quercetin, (b) EGFR-quercetin, (c) CCND1-quercetin, (d) CASP3-kaempferol, (e) MAPK8-kaempferol, and (f) PPARG-kaempferol.

The top two active compounds, quercetin and kaempferol, were selected to perform molecular docking with six relevant core genes (IL6, EGFR, CCND1, CASP3, MAPK8, and PPARG). The stability of the ligand-receptor complex was correlated with the binding energy. The lower the value of binding energy, the more stable the docking complex [21]. As shown in Table 2, IL6, EGFR, and CCND1 demonstrated strong binding to quercetin; similarly, CASP3, MAPK8, and PPARG demonstrated strong binding to kaempferol. A visual explanation of docking results analyzed the interaction between YPF-active ingredients and the potential targets of asthma (Figure 6). We found that quercetin had the best binding to EGFR with the binding energy -8.8 kcal/mol, and hydrophobic interactions and hydrogen-bonding interactions were the primary interaction forms.

## 4. Discussion

Asthma is a complicated respiratory tract disorder characterized by airway hyperresponsiveness and inflammation [22, 23]. The etiology of asthma is not completely clear, and it is generally believed that its occurrence is affected by environmental and genetic factors [24]. As the incidence of asthma continues to increase, the economic and social burden of the disease was relatively high [25]. YPF, as a traditional Chinese patent medicine, has been widely used for a long history. In an animal experiment [26], YPF has been shown to relieve airway inflammation in asthmatic mice. Therefore, we identified the potential key pathways based on network pharmacology to provide a theoretical foundation for subsequent investigations.

In the YPF's active ingredient-target network, a total of 60 target genes by 35 active components in the YPF were selected. The top two ingredients, quercetin and kaempferol, with the degree of 44 and 25, respectively, were chosen to perform molecular docking with six related core genes.

Quercetin is the principal representative of natural flavonoids and widely distributed in diverse food and plants [27]. A variety of biological functions of quercetin have been reported [28–30], such as anti-inflammation, antivirus, antioxidant activity, and immunoregulation effect. Mlcek et al. found that quercetin processes antiallergic effects, connected with inhibiting the histamine release from mast cells [31]. A pilot study [32] found that quercetin does great benefits for subjects suffering from asthma. Kaempferol belongs to the family of flavonoids, present in a lot of fruits and vegetables [33]. The properties of fighting free radicals, anti-inflammatory activity, and the anticancer effect of kaempferol have been reported [34]. Lin et al. found that kaempferol can regulate the transcriptional activity of FOXP3 and enhance Treg cells' suppressive activity [35]. Previous studies found that kaempferol ameliorates airway inflammation as well as antagonizes allergic reactions [36].

In this study, we constructed the PPI network; IL6, CASP3, EGFR, MAPK8, ESR1, CCND1, and PPARG were found to be the core genes of YPF in treating asthma. These genes are connected with host immunity, cell apoptosis, signal transduction, and cell cycle regulation. IL6 is a multifunctional cytokine and is generally related to eosinophil and neutrophil recruitment [37]. High blood levels of IL-6 were reported as a biomarker of asthma exacerbation [38]. In the execution of cell apoptosis, it has been suggested that CASP3 is a crucial enzyme. The upregulated expression of CASP3 led to apoptosis of epithelial cells in asthma [39]. EGFR, as well as MAPK8, played essential roles in airway inflammation, such as mucus production and secretion [40–42]. ESR1, one of the asthma candidate genes, is involved in pulmonary inflammation, causing a decline in lung function [43, 44].

The signal nucleotide polymorphism in CCND1 was linked with obesity [45]; meanwhile, it might participate in the process of asthma initiation and development [46]. PPARG is a central regulator in adipogenesis; PPARG-dependent transcription plays an essential role in regulating mitochondrial function [47, 48]. As is known to all, PPARG could promote adipocyte differentiation and adipogenesis [49]; moreover, obesity is a risk factor for asthma [50]. Consequently, PPARG may be a therapeutic target for obese asthma.

GO analysis indicated the diverse and complex synergistic effects of YPF and showed a few BP categories crucially involved with asthma. The top ten BP terms revealed that YPF could regulate the oxidative stress process and the immune response. There is an extraordinarily complex network between multiple cytokines and a number of signal-transduction pathways involved in the pathophysiological process of asthma [51]. KEGG enrichment results showed that various targets of YPF served crucial roles in asthma-related pathways, such as the TNF signaling pathway, PI3K-Akt signaling pathway, and IL-17 signaling pathway.

Further molecular docking results suggested that the docked small molecule ligands exhibited the lowest binding energy with a good affinity toward macromolecular protein receptors. All the binding energies were less than 6 kcal/mol. Quercetin was verified as the most potent binding activity with EGFR, while kaempferol had the most robust combination with PPARG.

## 5. Conclusion

In summary, this sufficiently thorough bioinformatic analysis provided plentiful testable hypotheses about the potential molecular mechanisms of YPF in treating asthma. It is indicated that the detailed action mechanisms of YPF in the treatment of asthma involve multiple ingredients, targets, and signaling pathways. Therefore, traditional Chinese medicine such as YPF could be considered as a supplementary regimen for future asthma therapy.

## Figures and Tables

**Table 1:** Potential pathways enriched by target genes.

Term	Description	GeneRatio	*q* value	GeneID
hsa04668	TNF signaling pathway	11/58	6.50*E* − 09	IL6/CASP3/CASP8/IKBKB/MAPK8/ICAM1/SELE/VCAM1/FOS/NFKBIA/IRF1
hsa04151	PI3K-Akt signaling pathway	14/58	9.64*E* − 07	CHRM1/CHRM2/HSP90AA1/CCND1/BCL2/IL6/MCL1/PRKCA/IKBKB/EGFR/RAF1/ERBB2/NOS3/IGF2
hsa04657	IL-17 signaling pathway	8/58	1.78*E* − 06	HSP90AA1/IL6/CASP3/CASP8/IKBKB/MAPK8/FOS/NFKBIA
hsa04659	Th17 cell differentiation	8/58	7.84*E* − 07	HSP90AA1/IL6/IKBKB/MAPK8/AHR/FOS/NFKBIA/HIF1A

**Table 2:** Molecular docking results.

Core genes (PDB ID)	Active ingredients	Binding energy (kcal/mol)
IL6 (1ALU)	Quercetin	-6.5
EGFR (1XKK)	Quercetin	-8.8
CCND1 (2W9F)	Quercetin	-7.1
CASP3 (3HOE)	Kaempferol	-7.1
MAPK8 (3PZE)	Kaempferol	-8.0
PPARG (2Q59)	Kaempferol	-8.4

## Data Availability

All the data analyzed in our study are included in the article and the supplementary materials.

## Supplementary Materials

Supplementary Materials The Supplementary Material (Supplementary Table S1: basic information of active ingredients in YPF) for the article can be found online at https://review.hindawi.com/f75cbaec-e20b-4ec7-b2ea-3d56e7ccfae0.
