# Supplementary material for: Molecular Mechanism of YuPingFeng in the Treatment of Asthma Based on Network Pharmacology and Molecular Docking Technology
Source: Comput Math Methods Med. 2022 Sep 5;2022:7364126. doi: 10.1155/2022/7364126 (PMC9467798; doi:10.1155/2022/7364126)
Supplement: Supplementary Materials — The Supplementary Material (Supplementary Table S1: basic information of active ingredients in YPF) for the article can be found online at https://review.hindawi.com/f75cbaec-e20b-4ec7-b2ea-3d56e7ccfae0. [file 7364126.f1.docx]

**Supplementary Table S1 Basic information of active ingredients in YPF**

| Mol ID | Ingredient | OB | DL | Degree | Herbs |
| --- | --- | --- | --- | --- | --- |
| MOL000098 | quercetin | 46.43 | 0.28 | 44 | HQ |
| MOL000422 | kaempferol | 41.88 | 0.24 | 25 | HQ |
| MOL000358 | beta-sitosterol | 36.91 | 0.75 | 15 | FF |
| MOL000378 | 7-O-methylisomucronulatol | 74.69 | 0.30 | 15 | HQ |
| MOL000173 | wogonin | 30.68 | 0.23 | 11 | FF |
| MOL000380 | (6aR,11aR)-9,10-dimethoxy-6a,11a-dihydro-6H-benzofurano[3,2-c]chromen-3-ol | 64.26 | 0.42 | 11 | HQ |
| MOL000354 | isorhamnetin | 49.60 | 0.31 | 10 | HQ |
| MOL000392 | formononetin | 69.67 | 0.21 | 9 | HQ |
| MOL000371 | 3,9-di-O-methylnissolin | 53.74 | 0.48 | 8 | HQ |
| MOL000049 | 3β-acetoxyatractylone | 54.07 | 0.22 | 7 | BZ |
| MOL011753 | 5-O-Methylvisamminol | 37.99 | 0.25 | 7 | FF |
| MOL000417 | Calycosin | 47.75 | 0.24 | 7 | HQ |
| MOL000011 | (2R,3R)-3-(4-hydroxy-3-methoxy-phenyl)-5-methoxy-2-methylol-2,3-dihydropyrano[5,6-h][1,4]benzodioxin-9-one | 68.83 | 0.66 | 6 | FF |
| MOL013077 | Decursin | 39.27 | 0.38 | 6 | FF |
| MOL000296 | hederagenin | 36.91 | 0.75 | 6 | HQ |
| MOL011740 | divaricatol | 31.65 | 0.38 | 5 | FF |
| MOL011747 | ledebouriellol | 32.05 | 0.51 | 5 | FF |
| MOL002644 | Phellopterin | 40.19 | 0.28 | 5 | FF |
| MOL003588 | Prangenidin | 36.31 | 0.22 | 5 | FF |
| MOL000239 | Jaranol | 50.83 | 0.29 | 5 | HQ |
| MOL000072 | 8β-ethoxy atractylenolide Ⅲ | 35.95 | 0.21 | 3 | BZ |
| MOL001941 | Ammidin | 34.55 | 0.22 | 3 | FF |
| MOL000359 | sitosterol | 36.91 | 0.75 | 3 | FF |
| MOL011730 | 11-hydroxy-sec-o-beta-d-glucosylhamaudol_qt | 50.24 | 0.27 | 2 | FF |
| MOL011732 | anomalin | 59.65 | 0.66 | 2 | FF |
| MOL011737 | divaricatacid | 87.00 | 0.32 | 2 | FF |
| MOL011749 | phelloptorin | 43.39 | 0.28 | 2 | FF |
| MOL001494 | Mandenol | 42.00 | 0.19 | 2 | FF |
| MOL000387 | Bifendate | 31.10 | 0.67 | 2 | HQ |
| MOL000442 | 1,7-Dihydroxy-3,9-dimethoxy pterocarpene | 39.05 | 0.48 | 2 | HQ |
| MOL000033 | (3S,8S,9S,10R,13R,14S,17R)-10,13-dimethyl-17-[(2R,5S)-5-propan-2-yloctan-2-yl]-2,3,4,7,8,9,11,12,14,15,16,17-dodecahydro-1H-cyclopenta[a]phenanthren-3-ol | 36.23 | 0.78 | 1 | HQ, BZ |
| MOL007514 | methyl icosa-11,14-dienoate |  |  | 1 | FF |
| MOL000211 | Mairin | 55.38 | 0.78 | 1 | HQ |
| MOL000379 | 9,10-dimethoxypterocarpan-3-O-β-D-glucoside | 36.74 | 0.92 | 1 | HQ |
| MOL000433 | FA | 68.96 | 0.71 | 1 | HQ |
